# Clinical characteristics of *Pneumocystis jirovecii* pneumonia in hemodialysis patients

**DOI:** 10.3389/fmed.2025.1521879

**Published:** 2025-04-07

**Authors:** Na Young Kim, So Jeong Kim, Yohwan Yeo, Taehee Kim, Ji-Young Park, Jeong-Hee Choi, Chang Youl Lee, Soo Jie Chung, Junghyun Kim

**Affiliations:** ^1^Department of Pulmonology and Allergy, Hallym University Dongtan Sacred Heart Hospital, Hwaseong, Republic of Korea; ^2^Lung Research Institute of Hallym University College of Medicine, Chuncheon, Republic of Korea; ^3^Department of Family Medicine, College of Medicine, Hallym University Dongtan Sacred Heart Hospital, Hwaseong, Republic of Korea; ^4^Department of Pulmonary, Allergy and Critical Care Medicine, Hallym University Kangnam Sacred Heart Hospital, Seoul, Republic of Korea; ^5^Division of Pulmonary, Allergy and Critical Care Medicine, Hallym University Sacred Heart Hospital, Anyang, Republic of Korea; ^6^Division of Pulmonary, Allergy and Critical Care Medicine, Hallym University Chuncheon Sacred Heart Hospital, Chuncheon, Republic of Korea

**Keywords:** end-stage renal disease, hemodialysis, *Pneumocystis pneumonia*, risk factors, *Pneumocystis carinii* (jiroveci)

## Abstract

**Background:**

Limited research exists on *Pneumocystis jirovecii* pneumonia (PJP) in hemodialysis (HD) patients. This retrospective study aimed to compare clinical features and outcomes of PJP in HD and non-HD patients.

**Methods:**

We retrospectively analyzed 10 HD PJP cases and 40 non-HD PJP cases which were matched propensity scoring. Criteria included respiratory symptoms, new pulmonary infiltrates, and positive *Pneumocystis* real-time PCR. HD PJP patients were excluded if they were taking immunosuppressants, receiving solid organ transplant, treatment for hematological or solid cancer, HIV infection, or *Pneumocystis jirovecii* colonization, which was not an actual infection.

**Results:**

No significant differences in symptoms and radiological findings were observed between HD and non-HD PJP cases. Fever was the main symptom in both groups, ground glass opacity was the main finding on CT, and there was no significant difference between the two groups in blood test results. Although the partial pressure of oxygen/fraction of inspired oxygen (PaO_2_/FiO_2_) ratio and SpO_2_/FiO_2_ ratio exhibited lower averages in the HD group, there was no significant difference between the two groups (*p* = 0.562 and 0.693, respectively). HD PJP patients exhibited delayed time to treatment marginally (5.3 ± 2.3 days vs. 3.0 ± 3.8 days, *p* = 0.051) and duration of treatment was longer than non-HD PJP (18.3 ± 3.2 days vs. 13.5 ± 7.5 days, *p* = 0.015). The length of stay, in-hospital mortality, and PJP-related death rate did not differ between HD PJP and non-HD PJP (*p* = 0.382, 0.724, and 1.000, respectively).

**Conclusion:**

Our study highlights that HD patients with PJP may encounter delays in diagnosis and treatment compared to non-HD PJP patients.

## Background

In patients undergoing hemodialysis (HD), infectious diseases represent a prevalent cause of death following cardiovascular disease. Furthermore, the burden of infections unrelated to HD, particularly pneumonia, constitutes a significant factor leading to mortality (1). The death rate attributable to pneumonia in HD patients is recognized to be 14-16 times higher than that in the general population (2). It has been suggested that HD patients may be vulnerable to opportunistic infections due to dysregulation of innate and adaptive immunity (3). For example, one study showed that Th1 was more prevalent than Th2 in HD patients (4), and another study found that CD4 and CD8 cells were below normal levels in HD patients (5). However, the exact mechanism remains undisclosed.

*Pneumocystis jirovecii* pneumonia (PJP) is an opportunistic pathogen that resides in the lungs and serves as a significant source of opportunistic infections in immunosuppressed patients (6). Well-known as a risk factor, lymphopenia due to acquired immune deficiencies such as human immunodeficiency virus (HIV) is acknowledged. However, approximately one-third of PJP can also manifest in non-HIV immunocompromised individuals, including those on long-term steroid therapy, undergoing anticancer treatment, dealing with autoimmune diseases, experiencing blood cancer, or undergoing organ transplantation (7).

Pneumonia in HD patients is known to exhibit a more severe clinical course compared to the general population and to be associated with a high risk of bloodstream infection (1). However, there is still insufficient research on aspects of atypical pneumonia, including PJP. According to one study, even in the HD group without transplantation, the risk of PJP was 29.9 times higher than that in the normal group. Nonetheless, most studies on the increased risk of developing PJP are limited to kidney transplant patients using immunosuppressants (8).

*P. jirovecii* is a microorganism that can be present even in healthy individuals and is known to cause infections such as pneumonia in people with reduced immunity. It has been suggested that the airborne human-to-human transmission of *P. jirovecii* in asymptomatic individuals may be a risk factor for infecting other vulnerable hosts (9). Meanwhile, *P. jirovecii* was detected in the sputum of 20.9% of HD patients, indicating a high *P. jirovecii* colonization rate. Among these, only 15.4% were using immunosuppressants for transplantation (10). Therefore, it can be assumed that HD itself may be a risk factor for PJP as a transmission source for high colonization rate. However, there is still no prior research on the possibility of HD as a risk factor for PJP or the clinical characteristics of PJP in HD patients.

Therefore, we conducted a retrospective review of PJP patients undergoing HD diagnosed at four university hospitals over the past 10 years, comparing their clinical features with those of PJP patients who did not undergo HD.

## Materials and methods

### Study population

We conducted a retrospective review of patients undergoing HD for end-stage renal disease (ESRD) among those diagnosed with PJP at four university hospitals: Hallym University Sacred Heart Hospital, Anyang, South Korea, Hallym University Gangnam Sacred Heart Hospital, Seoul, South Korea, Hallym University Chuncheon Sacred Heart Hospital, Chuncheon, South Korea, and Hallym University Dongtan Sacred Heart Hospital, Hwaseong, South Korea, from November 2005 to June 2023. Current study enrolled patients diagnosed with PJP who were admitted to the hospital, excluding those meeting the following conditions: (1) taking immunosuppressants, (2) receiving solid organ transplants, (3) being treated for blood cancer or solid cancer, (4) having HIV infection, and (5) exhibiting *P. jirovecii* colonization rather than true infection. We collected information as follows: clinical information, including age, gender, underlying disease, and PJP symptoms of both patient and control groups; details about the specimen from which *P. jirovecii* positivity was obtained (sputum, bronchial washing, BAL specimen); imaging information, such as chest X-ray and CT; details about PJP treatment, time to treatment, treatment medication and duration, number of days of hospitalization, discharge, and death. This study was approved by the Institutional Review Board at each hospital (HDT 2022-10-013). Because the study was retrospective in nature, the requirement for patient consent was exempted.

### Diagnosis of PJP

Two pulmonologists (NYK and SJC) reviewed the medical records, ensuring that all three of the following criteria were met for diagnoses of PJP: (1) clinical symptoms (dyspnea, cough, and/or fever) were apparent (2) positive *P. jirovecii* real-time PCR and/or indirect immunofluorescence results from sputum, bronchial washing, or bronchoalveolar lavage fluid, and (3) radiographic features indicative of PJP included bilateral interstitial infiltrates, ground glass opacities, reticular opacities, or septal thickening was observed. Positive microbiological tests without concurrent clinical manifestations consistent with respiratory infection were not considered indicative of PJP.

### Statistical analysis

Categorical variables use the chi-square test or Fisher’s exact test, continuous variables use Student’s *t*-test or Mann-Whitney test. Propensity score matching was used to obtain balance between the HD and non-HD groups. In the total population, we used 1:4 nearest-neighbor matching by age and sex. All statistical analyzes used R version 4.3.1 (R Foundation for Statistical Computing, Vienna, Austria) and statistical significance was set at *P* < 0.05.

## Results

**FIGURE 1:**
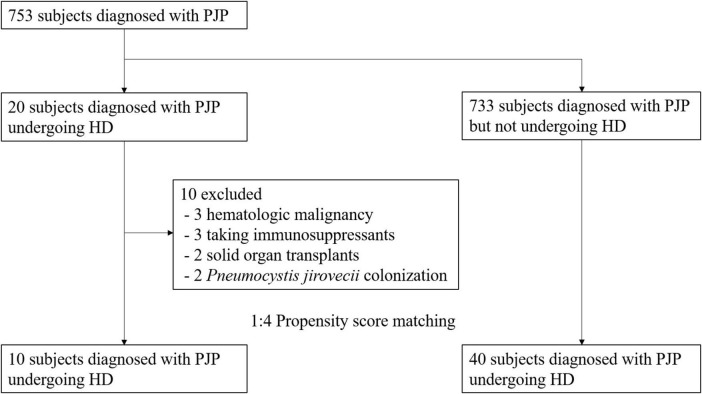
Patients flowchart.

Among the 753 patients diagnosed with PJP, 20 were undergoing dialysis, while the 733 were PJP patients not receiving dialysis. Ten patients with PJP were excluded from the study due to predefined exclusion criteria, including three cases of hematologic malignancy, three patients receiving immunosuppressants, two with a history of solid organ transplants, and two instances of *P. jirovecii* colonization (Figure 1). The remaining 10 patient were subjected to propensity matching, selecting non-dialysis PJP patients at a 1:4 ratio by age and sex (Figure 1).

### Baseline characteristics of patients with diagnosed PJP

The baseline characteristics of the study subjects were categorized into HD cases (*n* = 10) and non-HD groups (*n* = 40) and summarized in Table 1. In the HD group, 80% were male, with an average age of 72.5 ± 12.2 years (Mean ± Standard Deviation), which was not statistically different from the non-HD group (82.5% male and age 73.2 ± 10.5 years). Additionally, there were no significant differences in body mass index (BMI) and smoking status between the two groups. Most comorbidities showed no significant differences, but the rate of chronic liver disease was higher in the HD group, while the rates of adrenal insufficiency and thyroid disease were higher in the non-HD group. When examining the etiology of nephropathy in the HD group, hypertensive (60%) and diabetic nephropathy (40%) were the most common causes.

**TABLE 1:** Demographic and clinical characteristics of study patients.

	HD (*n* = 10)	Non-HD (*n* = 40)	*P*-value
Male sex	8 (80.0)	33 (82.5)	1.000
Age, years	72.5 ± 12.2	73.2 ± 10.5	0.861
BMI, kg/m^2^	23.4 ± 4.0	22.5 ± 3.3	0.512
**Smoking status**			
Ever smoker	2 (20.0)	5 (12.5)	0.616
Never smoker	8 (80.0)	35 (87.5)	
**Comorbidities**			
Chronic kidney disease	10 (100)	35 (87.5)	0.569
Chronic liver disease	3 (30.0)	0 (0.0)	0.006
Cerebral vascular disease	1 (10.0)	2 (5.0)	0.496
Hypertension	8 (80.0)	17 (42.5)	0.077
Diabetes mellitus	5 (50.0)	10 (25.0)	0.143
Adrenal insufficiency	7 (70.0)	39 (97.5)	0.022
Heart failure	10 (100.0)	38 (95.0)	1.000
Chronic airway disease	0 (0.0)	5 (12.5)	0.569

Data are presented as n (%) or mean ± standard deviation. HIV, human immunodeficiency virus; IgA, immunoglobulin A; PCKD, polycystic kidney disease.

### Clinical manifestations of patients with PJP

**FIGURE 2:**
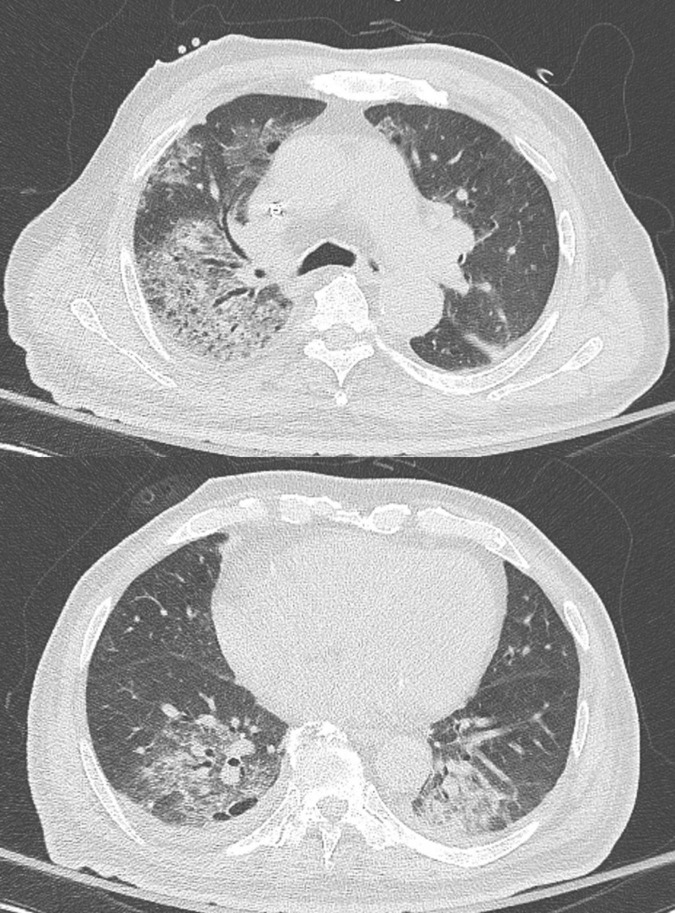
A low dose chest CT shows multifocal peribronchial pneumonic consolidations and ground-glass opacities in right upper lobe and both lower lobes, as well as bilateral pleural effusion.

The clinical symptoms of PJP patients were categorized into two groups (HD vs. non-HD) and are summarized in Table 2. There were no statistically significant differences in symptoms between the two groups. However, in the HD group, fever and cough were the most common symptoms, while in the non-HD group, fever was also predominant, followed by dyspnea and cough. Regarding radiological findings, ground glass opacity was the most common in both groups (HD 100% vs. non-HD 89.2%), and pleural effusion appeared to be more dominant in the HD group (HD 60.0% vs. non-HD 48.6%) (Figure 2). However, none of these findings showed a statistically significant difference. Blood test results, including white blood cell profile, CRP, LDH, and albumin, showed no significant differences between the two groups. For 1,3 β-D-glucan, the levels were 100.0 ± 71.4 in the HD group and 495.6 ± 458.9 in the non-HD group, with no significant difference between the two groups (*p* = 0.435). Although the PaO_2_/FiO_2_ ratio and SpO_2_/FiO_2_ ratio exhibited lower averages in the HD group, there was no significant difference between the two groups (*p* = 0.562, *p* = 0.693, respectively).

**TABLE 2:** Comparison of clinical manifestations of PJP between HD and non-HD patients.

	HD (*n* = 10)	Non-HD (*n* = 40)	*P*-value
**Clinical symptoms**			
Fever	6 (60.0)	22 (55.0)	1.000
Cough	4 (40.0)	11 (27.5)	0.462
Dyspnea	1 (10.0)	15 (37.5)	0.138
Hemoptysis	1 (10.0)	1 (2.5)	0.363
Chest discomfort	1 (10.0)	1 (2.5)	0.363
**Radiological manifestation**			
Ground glass opacity	10 (100.0)	33 (89.2)	0.564
Consolidation	5 (50.0)	12 (32.4)	0.460
Pleural effusion	6 (60.0)	18 (48.6)	0.724
Pneumothorax	0 (0.0)	1 (2.7)	1.000
**Laboratory parameters**			
WBC, /μL	10855.0 ± 6289.9	9043.8 ± 10699.7	0.495
PMN, ×10^3^/μL	82.4 ± 9.1	80.7 ± 11.5	0.622
Lymphocyte, ×10^3^/μL	8.3 ± 4.8	9.7 ± 6.2	0.479
ANC, /μL	9073.0 ± 5745.7	6129.5 ± 4748.0	0.160
CRP, g/dL	76.8 ± 59.4	86.7 ± 67.7	0.653
LDH, IU/L	356.0 ± 209.0	472.4 ± 488.1	0.267
Albumin, g/dL	3.1 ± 0.6	3.0 ± 0.7	0.883
PF ratio	163.3 ± 92.3	197.4 ± 145.4	0.562
SF ratio	312.5 ± 125.4	334.2 ± 118.7	0.693
1,3 β-D-glucan, pg/mL	100.0 ± 71.4	495.6 ± 458.9	0.435

For clinical symptoms and radiological manifestations, the data are presented as the number of patients with the percentage in parentheses. For laboratory parameters, values are expressed as mean ± standard deviation. ANC, absolute neutrophil count; CRP, C-reactive protein; LDH, lactate dehydrogenase; PJP, *Pneumocystis jirovecii* pneumonia; PF ratio, PaO_2_/FiO_2_ ratio; PMN, Polymorphonuclear neutrophil; SF ratio, SpO_2_/FiO_2_ ratio; WBC, white blood cell.

### Treatment and outcome of patients with PJP

The treatment progress of PJP patients was categorized into two groups: HD and non-HD and is summarized in Table 3. Compared to non-HD PJP patients, the time to treatment in HD PJP patients was delayed, although not statistically significant (5.3 ± 2.3 vs. 3.0 ± 3.8 days, *p* = 0.051), and the treatment period was significantly longer (18.3 ± 3.2 vs. 13.5 ± 7.5 days, *p* = 0.015). Notably, three patients in the HD group resulted in death without treatment due to delayed differential diagnosis, a circumstance absent in the non-HD group. Trimethoprim/sulfamethoxazole (TMP/SMX) was the primary therapy used in both groups. Although the use rate of TMP/SMX in the HD group appears to be lower than that in the non-HD group, this was a phenomenon seen because there were 3 cases of death in the HD group without treatment. There was no significant difference in the use of adjunctive steroids or the type of oxygen therapy between the two groups.

**TABLE 3:** Initiation and approaches to treatment in patients with PJP.

	HD (*n* = 10)	Non-HD (*n* = 40)	*P*-value
Time to diagnosis, days	14.1 ± 15.3	10.7 ± 17.0	0.550
Time to treatment, days	5.3 ± 2.3	3.0 ± 3.8	0.051
No treatment	3 (30.0)	0 (0.0)	0.007
**Antibiotics**			
TMP/SMX	6 (60.0)	38 (100.0)	0.001
Duration of treatment	18.3 ± 3.2	13.5 ± 7.5	0.015
Primaquine + Clindamycin	1 (10.0)	2 (5.0)	0.496
Duration of treatment	3.0 ± NA	1.5 ± 0.7	NA
**Adjunctive steroid**	6 (60.0)	22 (55.0)	1.000
**Oxygen therapy**	4 (40.0)	28 (70.0)	0.162
Low flow oxygen therapy	2 (20.0)	6 (15.0)	1.000
High flow oxygen therapy	1 (10.0)	8 (20.0)	0.782
Mechanical ventilation	1 (10.0)	14 (35.0)	0.247

Data are presented as n (%) or mean ± standard deviation. PJP, *Pneumocystis jirovecii* pneumonia; TMP/SMX, Trimethoprim/Sulfamethoxazole.

The outcomes of PJP patients were divided into HD and non-HD groups and are summarized in Table 4. While the length of stay in the HD group was longer than that in the non-HD group, there was no statistically significant difference (*p* = 0.382). In-hospital mortality (40.0 vs. 52.5%, *p* = 0.724), 28-day mortality (40.0 vs. 35.0%, *p* = 1.000), and PJP-related death (30.0 vs. 32.5%, *p* = 1.000) also exhibited no significant differences between the two groups.

**TABLE 4:** Clinical outcomes and mortality of patients with PJP.

	HD (*n* = 10)	Non-HD (*n* = 40)	*P*-value
**Hospital outcome**			
Length of stay	41.5 ± 33.6	31.3 ± 21.9	0.382
In-hospital mortality	4 (40.0)	21 (52.5)	0.724
28-day mortality	4 (40.0)	14 (35.0)	1.000
**Death related to PJP**	3 (30.0)	13 (32.5)	1.000

Data are presented as n (%) or mean ± standard deviation. PJP, *Pneumocystis jirovecii* pneumonia.

## Discussion

Epidemiology on the occurrence and clinical presentation of PJP in HD patients are exceedingly rare. In an observational study conducted in Denmark, the incidence rate of hospitalization of HD patients due to PJP was over 30 times higher than that in the general population (43.1 per 100,000 person-years vs. 1.43 per 100,000 person-years) (8). However, this study only assessed incidence rates and did not examine the clinical aspects of PJP. There was a total of 5 case reports, and mortality was reported in 80% of the 5 cases (11–15). However, it was confirmed that all of them had taken steroids or other immunosuppressants. Consequently, it cannot be conclusively stated that these cases strictly represent the clinical picture of PJP as a risk factor for HD alone. In our study, in-hospital mortality in HD patients was 40% and death related to PJP was 30%, which was lower than previous studies, but showed no significant difference from non-HD PJP.

In this study, we compared the clinical aspects of PJP in HD patients, excluding patients who were concurrently taking immunosuppressants, were HIV positive, or were receiving cancer treatment, with non-HD PJP patients. In comparison to the control group, PJP patients on HD exhibited no symptoms or laboratory characteristics comparable to the controls. In HD PJP, compared to non-HD, fever and cough were the main symptoms rather than dyspnea, and GGO was dominant in both groups. Meanwhile, it was unusual to see pleural effusion in around 50% of cases in both groups. Previous studies have also reported that pleural effusion is rare in PJP (16). Pleural effusion is particularly common in HD patients due to volume overload, but the possibility that this pleural effusion may be caused by PJP should not be overlooked.

In actual practice guidelines, TMP/SMX is recommended as first-line treatment without distinguishing between HD and non-HD PJP. In this study, TMP/SMX was mostly used as a treatment drug with no difference between HD and non-HD. Among the HD patients, one patient received primaquine + clindamycin (PQ/CLDM) as the initial choice due to concerns about side effects and volume overload associated with TMP/SMX. In contrast, among the non-HD patients, two patients opted for PQ/CLDM as a second-line treatment, having initially received TMP/SMX but experienced side effects. The time to treatment of TMP/SMX was marginally longer in the HD group than in the control group (5.3 ± 2.3 vs. 3.0 ± 3.8 days, *p* = 0.051). In the three cases of death, there was a possibility that death occurred before PJP diagnosis, resulting in delayed diagnosis and treatment. Additionally, the total treatment period was longer in the HD group (18.3 ± 3.2 vs. 13.5 ± 7.5 days, *p* = 0.015). These showed the possibility that it took time for clinicians to suspect and diagnose PJP because HD itself is not yet known to be a risk factor for PJP. Therefore, it is very important for physicians to promptly diagnose and treat HD patients with PJP.

It can be inferred from previous studies that HD can be a risk factor for PJP. One hypothesis for how HD may be a risk factor for PJP involves possible immune dysfunction in patients with HD. It is well-established that cluster of differentiation 4 positive T cells (CD4^+^ T cells) play a pivotal role in PJP infection (17). In HIV patients, a recognized risk factor for PJP, a decrease in peripheral blood CD4^+^ T cells was observed, known to elevate the risk of PJP (18). Furthermore, CD4^+^ T cells decline was also observed in non-HIV patients taking immunosuppressants (19). In HD patients, Type 1 T helper cells (Th1) exhibited a more polarized cell-mediated immunity than Type 2 T helper cells (Th2) (4). In addition, it was confirmed that HD patients experience early maturation of T cell immunity, high turnover and apoptosis (20), and a decrease in CD4^+^ T cells and CD8^+^ T cells (5). There is a possibility that HD patients may be susceptible to PJP due to a lower level and dysfunctional CD4^+^ T cells. Secondly, the high *P. jirovecii* colonization in HD patients is likely to be a risk factor for PJP. In a previous study, *P. jirovecii* PCR was detected in 20.9% of sputum samples from HD patients (10). It has been suggested that *P. jirovecii* colonization in asymptomatic individuals may serve as a potential source of infection for vulnerable hosts (9). Therefore, there is a possibility that HD itself increases the risk of *P. jirovecii* colonization and thus increases the risk of PJP. However, in our study, PJP in HD patients showed no significant differences compared to non-HD in terms of outcomes such as length of stay and mortality. A larger prospective study is needed in the future to determine whether HD will be a risk factor for PJP.

This study has the following limitations. Firstly, since we retrospectively distinguished between HD and non-HD cases in which PJP had already occurred, we couldn’t directly compare the incidence rate of PJP caused by HD with non-HD cases. Secondly, although propensity matching was performed for sex and age between PJP in HD and non-HD PJP cases, other factors could not be matched, so the possibility of other compounding factors, aside from HD, cannot be ruled out. Thirdly, there is a limitation in that the number of subjects is relatively small. Nevertheless, this is the only study to comprehensively examine the characteristics of HD PJP by comparing this very rare disease in HD patients with non-HD PJP, using propensity matching. Fourthly, PJP was diagnosed through *P. jirovecii* PCR, with indirect fluorescent antibody staining rarely performed, and direct microscopic evaluations such as Giemsa, Periodic Acid-Schiff (PAS), and Toluidine Blue O (TBO) not conducted, thereby limiting the diagnostic accuracy.

## Conclusion

Our study found that there were no significant differences in outcomes such as mortality in HD patients with PJP compared to patients without HD, but that there may be delays in diagnosis and treatment.

## Data Availability

The original contributions presented in the study are included in the article, further inquiries can be directed to the corresponding authors.
